# Ezetimibe Attenuates Oxidative Stress and Neuroinflammation via the AMPK/Nrf2/TXNIP Pathway after MCAO in Rats

**DOI:** 10.1155/2020/4717258

**Published:** 2020-01-03

**Authors:** Jing Yu, Wen-na Wang, Nathanael Matei, Xue Li, Jin-wei Pang, Jun Mo, Sheng-pan Chen, Ji-ping Tang, Min Yan, John H. Zhang

**Affiliations:** ^1^Department of Anesthesiology, The Second Affiliated Hospital, Zhejiang University School of Medicine, Hangzhou, Zhejiang 310009, China; ^2^Department of Physiology and Pharmacology, School of Medicine, Loma Linda University, Loma Linda, CA 92354, USA; ^3^Department of Ophthalmology, University of Southern California, Los Angeles, CA 90007, USA; ^4^Department of Neurosurgery and Anesthesiology, Loma Linda University Medical Center, Loma Linda, CA 92354, USA

## Abstract

Oxidative stress and neuroinflammation play essential roles in ischemic stroke-induced brain injury. Previous studies have reported that Ezetimibe (Eze) exerts antioxidative stress and anti-inflammatory properties in hepatocytes. In the present study, we investigated the effects of Eze on oxidative stress and neuroinflammation in a rat middle cerebral artery occlusion (MCAO) model. One hundred and ninety-eight male Sprague-Dawley rats were used. Animals assigned to MCAO were given either Eze or its control. To explore the downstream signaling of Eze, the following interventions were given: AMPK inhibitor dorsomorphin and nuclear factor erythroid 2-related factor 2 (Nrf2) siRNA. Intranasal administration of Eze, 1 h post-MCAO, further increased the endogenous p-AMPK expression, reducing brain infarction, neurologic deficits, neutrophil infiltration, microglia/macrophage activation, number of dihydroethidium- (DHE-) positive cells, and malonaldehyde (MDA) levels. Specifically, treatment with Eze increased the expression of p-AMPK, Nrf2, and HO-1; Romo-1, thioredoxin-interacting protein (TXNIP), NOD-like receptor protein 3 (NLRP3), Cleaved Caspase-1, and IL-1*β* were reduced. Dorsomorphin and Nrf2 siRNA reversed the protective effects of Eze. In summary, Eze decreases oxidative stress and subsequent neuroinflammation via activation of the AMPK/Nrf2/TXNIP pathway after MCAO in rats. Therefore, Eze may be a potential therapeutic approach for ischemic stroke patients.

## 1. Introduction

Stroke accounts for 10% of all deaths worldwide [1]. The pathophysiology of stroke is composed of complex sequelae of cellular processes: oxidative stress, apoptosis, blood-brain barrier disruption, and inflammation [2–7]. Although the majority of ischemic strokes occur from embolic arterial occlusion, oxidative stress and neuroinflammation play significant roles in transient ischemic stroke and the reperfusion process [8, 9]. For example, neuroinflammatory responses to ischemic stroke are characterized by astrocyte activation and microglial resident, peripheral leukocyte infiltration, and proinflammatory mediator release. Moreover, infiltrated neutrophils and activated microglia produce free radicals and oxidants that damage the central nervous system tissue, leading to long-term disabilities and death in stroke patients [10]. Therefore, developing a protective strategy against oxidative stress and subsequent neuroinflammation may be an effective approach for the treatment of ischemic stroke patients.

Ezetimibe (Eze) is a new lipid-lowering agent that inhibits Niemann-Pick disease type C1-like 1- (NPC1L1-) dependent cholesterol absorption [11, 12]; however, studies have shown Eze to exert pleiotropic effects independent of NPC1L1 [13–15]. For example, we have previously demonstrated that intranasal administration of Eze attenuated neuronal apoptosis through the activation of AMPK-dependent autophagy after MCAO in rats [16]. In a rat liver ischemia/reperfusion model, Eze therapeutically exerted antioxidation effects by modulating glutathione and glutathione peroxidase [14]. In an Alzheimer mouse model, researchers reported that treatment with Eze reduced the memory dysfunctions associated with dementia [17]. Of importance, a randomized and placebo-controlled clinical study reported that treatment with Eze prevented the progression of the deleterious symptoms associated with acute stroke [18]. Lastly, in hepatocyte mouse models, studies have shown that the anti-inflammatory effects of Eze were dependent on AMPK autophagic induction and NLRP3 inflammasome inhibition [19, 20].

Mechanistically, AMPK phosphorylation promotes the activation of the master antioxidant regulator, nuclear factor erythroid 2-related factor 2 (Nrf2) [21], and reduces free radicals by increasing heme oxygenase 1 (HO-1), a downstream factor of Nrf2, which decreases proinflammatory cytokines [22]. Linking oxidative stress to inflammation in ischemic stroke, the inhibition of thioredoxin-interacting protein (TXNIP) was shown to decrease the activation of inflammasome-dependent pathways [23–25]. For example, in an acute cerebral ischemic injury model, activation of Nrf2 attenuated TXNIP and NOD-like receptor protein 3 (NLRP3) inflammasomes [26]. Taken together, Eze exerts its pleiotropic effects through activation of Nrf2 via AMPK-dependent pathways [20].

Therefore, in the current study, we assessed the hypothesis that intranasal administration of Eze may attenuate oxidative stress and neuroinflammation in a rat model of MCAO via the AMPK/Nrf2/TXNIP pathway.

## 2. Materials and Methods

### 2.1. Animals

All experiments were approved by the Institutional Animal Care and Use Committee of Loma Linda University in accordance with the NIH Guide for the Care and Use of Laboratory Animals (NIH Publications No. 8023, revised 1978) and the ARRIVE2009 Guidelines for Reporting Animal Research [27]. A total of 198 adult male Sprague-Dawley rats (260-280 g) were obtained from the Experimental Animal Center of Loma Linda University. Rats were housed in a controlled humidity and temperature room with a 12 h light/dark cycle and free access to water and food.

### 2.2. MCAO Model

The transient MCAO model was used in male Sprague-Dawley rats as previously described [28]. Briefly, anesthesia was induced intraperitoneally with a mixture of ketamine (80 mg/kg, K2573; Sigma-Aldrich, St. Louis, MO, USA) and xylazine (10 mg/kg, X1126; Sigma-Aldrich, St. Louis, MO, USA). Next, atropine was administered (0.1 mg/kg) subcutaneously. The depth of anesthesia was checked by pinch-paw reflex. The right common carotid artery (CCA), internal carotid artery (ICA), and external carotid artery (ECA) were surgically exposed. The ECA was ligated, and a 4–0 nylon suture with a silicon tip was then inserted through the ECA stump into the ICA, occluding the MCA, approximately 18 to 22 mm from the insertion point. After 2 h of MCAO, the suture was removed to begin reperfusion. Sham rats underwent the same protocol without occlusion of the MCA.

### 2.3. Experimental Design

**Figure 1:**
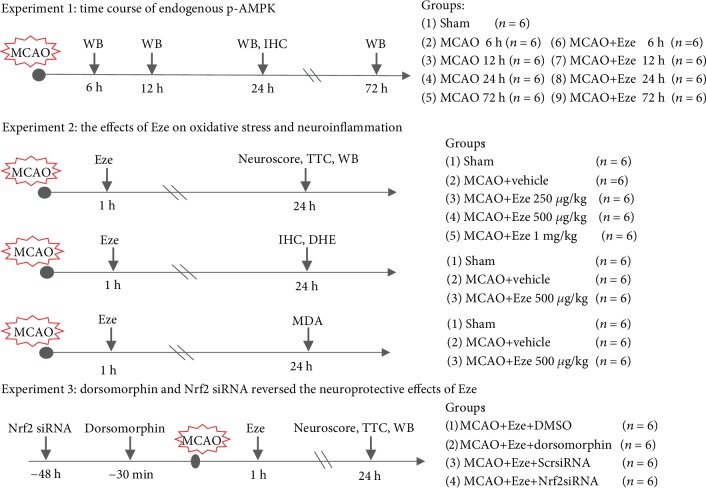
Experimental design and animal groups. DHE: dihydroethidium; DMSO: dimethyl sulfoxide; dorsomorphin: AMPK inhibitor; Eze: Ezetimibe; IHC: immunohistochemistry; MCAO: middle cerebral artery occlusion; MDA: malonaldehyde; Scr siRNA: scramble siRNA; TTC: 2,3,5-triphenyltetrazolium chloride; WB: western blot.

Animals were divided into groups for three experimental studies in a randomized fashion by generating random numbers using Excel, and experiments were performed in a blinded manner (Figure 1): the experimental groups and sample size are listed in Table 1.

#### 2.3.1. Experiment 1

To determine the time course of endogenous AMPK and phosphorylated AMPK (p-AMPK) after MCAO or MCAO+Eze, 54 rats were randomly divided into 9 groups (*n* = 6 per group): sham, MCAO 6 h, MCAO 12 h, MCAO 24 h, MCAO 72 h, MCAO+Eze 6 h, MCAO+Eze 12 h, MCAO+Eze 24 h, and MCAO+Eze 72 h. Western blot analysis was used to detect the expression of p-AMPK in the ipsilateral/right hemisphere of each group.

#### 2.3.2. Experiment 2

To evaluate the neuroprotective effects of intranasal administration of Eze at 1 h after MCAO, 30 rats were randomly assigned into the following five groups (*n* = 6 per group): (1) sham group, (2) MCAO+vehicle (10% dimethyl sulfoxide (DMSO) in phosphate-buffered saline (PBS)), (3) MCAO+Eze (250 *μ*g/kg), (4) MCAO+Eze (500 *μ*g/kg), and (5) MCAO+Eze (1 mg/kg). Infarction volume, modified Garcia, and beam walking scores were assessed at 24 h after MCAO (*n* = 6 per group). After 2,3,5-triphenyltetrazolium chloride (TTC) staining, the ipsilateral/right brain samples were collected for additional western blots (*n* = 6 per group). According to our infarction volume and neurobehavioral results, Eze at a dose of 500 *μ*g/kg had the highest efficacy and was used for the subsequent experiments.

To explore the effects of Eze treatment on neutrophil infiltration and microglia/macrophage activation at 24 h after MCAO, 18 rats were randomly divided into sham, MCAO+vehicle, and MCAO+Eze 500 *μ*g/kg groups (*n* = 6 per group). Immunofluorescence staining of myeloperoxidase (MPO) and Iba-1 was performed, and quantitative analyses of MPO and Iba-1-positive cells were counted in the ischemic penumbra at 24 h after MCAO. The expression of MPO and Iba-1 among the three groups was measured by western blot at 24 h after MCAO.

To explore the effects of Eze treatment on oxidative stress at 24 h after MCAO, another 18 rats from sham, MCAO+vehicle, and MCAO+Eze 500 *μ*g/kg were used to measure malonaldehyde (MDA) levels (*n* = 6 per group). Dihydroethidium (DHE) staining was performed, and the number of DHE-positive cells was counted in the ischemic penumbra at 24 h after MCAO (shared with the immunofluorescence-stained samples). The expression of Romo-1 among the three groups was measured by western blot at 24 h after MCAO.

#### 2.3.3. Experiment 3

To explore the underlying mechanisms of Eze-mediated antioxidation and anti-inflammatory effects after MCAO, dorsomorphin, a selective AMPK inhibitor, was administered intracerebroventricularly (i.c.v.) 30 min before MCAO; Nrf2 small interfering RNA (Nrf2 siRNA) was administered i.c.v. at 48 h before MCAO, followed by administration of Eze (500 *μ*g/kg) at 1 h after MCAO. Rats were randomly divided into seven groups (*n* = 6 per group): sham, MCAO+vehicle, MCAO+Eze, MCAO+Eze+DMSO, MCAO+Eze+dorsomorphin, MCAO+Eze+scramble siRNA (Scr siRNA), and MCAO+Eze+Nrf2 siRNA. Ipsilateral brain samples were collected for TTC staining and western blots at 24 h after MCAO (sham, MCAO+vehicle, and MCAO+Eze were shared with experiment 2; *n* = 6 per group). Infarction volume, neurobehavioral tests, and western blot analyses were performed at 24 h after MCAO.

Additionally, to evaluate the effects of AMPK inhibition via dorsomorphin, 24 rats were randomly divided into four groups: naive+DMSO, naive+dorsomorphin, naive+Eze+DMSO, and naive+Eze+dorsomorphin. The expression of phosphorylated AMPK was evaluated by western blot.

### 2.4. Intranasal Administration of Eze

Intranasal administration was performed as previously described [29]. Briefly, rats were treated 1 h after MCAO with DMSO or Eze (250 *μ*g/kg, 500 *μ*g/kg, and 1 mg/kg dissolved in 10%DMSO, purity ≥ 98%, SML-1629, Sigma-Aldrich, St. Louis, MO, USA) in a supine position under 2% isoflurane anesthesia. A total volume of 25 *μ*l was delivered into the bilateral nares, alternating one naris at a time, every 5 min over a period of 20 min.

### 2.5. Intracerebroventricular Injection

Intracerebroventricular (i.c.v.) administration was performed as previously described [28]. Briefly, rats were placed in a stereotaxic apparatus under 2.5% isoflurane anesthesia. A scalp incision was made along the midline, and a 1 mm burr hole was drilled into the skull. The stereotactic i.c.v. injection site was relative to the bregma: anteroposterior 1 mm, right lateral 1.5 mm, and depth 3.5 mm. The AMPK-specific inhibitor, dorsomorphin (0.1 *μ*mol, purity ≥ 98%, P5499, Sigma-Aldrich, St. Louis, MO, USA), was dissolved in 20% DMSO in PBS, and 10 *μ*l was delivered into the ipsilateral ventricle with a Hamilton syringe (Microliter 701; Hamilton Company, USA) 30 min before MCAO [30]. The same volume of DMSO was used as a negative control. Nrf2 siRNA (SR508224; OriGene, Rockville, MD, USA) and scrambled siRNA (SR30004; OriGene) were prepared at 500 pmol in RNAse free suspension buffer and administered (5 *μ*l of the siRNAs) 48 h before MCAO [31]. Lastly, the burr hole was sealed with bone wax, and the dissection was sutured.

### 2.6. Neurobehavioral Function Assessment

Neurobehavioral function was assessed with the modified Garcia and beam walking tests by an independent, blinded researcher at 24 h after MCAO, as previously described [32]. To understand the effect of neuronal lesions on sensorimotor areas, the modified Garcia test was used to measure hemiplegia, motor performance deficits, and abnormal postures [33]. The modified Garcia scoring system consisted of 6 tests covering spontaneous activity, symmetry in the movement of four limbs, forepaw outstretching, climbing, body proprioception, and response to vibrissae touch, with a maximum score of 18, higher scores indicating better performance. In addition, to better asses cortical motor injury, the beam walking test was used to measure dysfunction in memory, motivation, attention, somatomotor, and locomotor functions [34]. The beam walking test was performed with a 0-5-point scale as previously described [35].

### 2.7. Cerebral Infarction Volume Assessment

Under deep anesthesia, animals were perfused with cold PBS (0.1 M, pH 7.4) as previously described [36]. Brains were removed and coronally sliced into 2 mm thick sections. Brain slices were incubated in 2% 2,3,5 triphenyltetrazolium chloride (TTC, Sigma-Aldrich, St. Louis, MO, USA) for 15 min at 37°C. The infarcted brain tissue appeared white, whereas the noninfarcted region appeared red. The infarct and total hemispheric areas of each slice were measured using ImageJ (ImageJ 1.5; NIH, Bethesda, MD, USA). The area of each slice was calculated using the following formula: ((area of contralateral − *area* *of* *noninfarcted* *ipsilateral* *tissue*)/2∗(*area* of *contralateral*))∗100%. The area was calculated for each slice, and the average was taken to represent the percentage of infarcted area for that animal [37, 38].

### 2.8. Immunofluorescence Staining

Twenty-four hours after MCAO, under deep anesthesia, rats were perfused with ice-cold PBS and then 10% formalin. The brains were removed and fixed in formalin and then dehydrated with 30% sucrose. Next, brain samples were snap-frozen and cut into 10 *μ*m thick coronal sections using a cryostat (LM3050S; Leica Microsystems, Bannockburn, Germany). Immunofluorescence staining was performed as previously described [39]. Briefly, brain samples were incubated overnight at 4°C with primary antibodies including anti-Iba-1 (1 : 100, Abcam, ab5076) and anti-MPO (1 : 500, Abcam, ab65871). The sections were then incubated with the appropriate fluorescence-conjugated secondary antibodies (1 : 200, Jackson ImmunoResearch) for 1 h at room temperature and then visualized with a fluorescence microscope (DMi8, Leica Microsystems, Germany).

### 2.9. Measurement of Oxidative Stress

#### 2.9.1. MDA Assay

Malonaldehyde (MDA), as an oxidative damage marker, was determined using the MDA assay kits (MAK805, Sigma-Aldrich, St. Louis, MO, USA) as per the manufacturer's protocols.

#### 2.9.2. DHE Staining

Dihydroethidium (DHE) staining was performed as previously described [40]. Briefly, 10 *μ*m thick frozen brain sections were incubated with 2 *μ*mol/l fluorescent dye DHE (D1168, Thermo Fisher Scientific, Waltham, MA, USA) at 37°C for 30 min in a humidified chamber and protected from light. The DHE-positive cells were observed under a fluorescence microscope (DMi8, Leica Microsystems, Germany), and the positive cells were counted by using ImageJ software (ImageJ 1.5; NIH, Bethesda, MD, USA).

### 2.10. Western Blot Analysis

After TTC staining at 24 h after MCAO, brain slices were separated into the contralateral and ipsilateral hemispheres, flash frozen in liquid nitrogen, and then stored at −80°C freezer. Western blot was performed as previously described [41]. Nuclear proteins were extracted from tissue homogenates using a nuclear extraction kit (ab113474, Abcam, Cambridge, MA, USA) according to the manufacture's protocol. Equal amounts of protein samples from the ipsilateral hemispheres were separated by 10% SDS-PAGE and transferred onto nitrocellulose membranes. After blocking with 5% nonfat milk at 20–25°C for 1 h, samples were incubated overnight at 4°C with primary antibodies against AMPK (1 : 1000, CST, 5832), phosphorylated AMPK (p-AMPK, 1 : 1000, CST, 2535), Nrf2 (1 : 1000, Abcam, ab31163), HO-1 (1 : 1000, Abcam, ab13248), TXNIP (1 : 500, Proteintech, 18243-1-AP), NLRP3 (1 : 500, Abcam, ab214185), Caspase-1 (1 : 500, NOVUS, NBP1-45433), Interleukin- (IL-) 1*β* (1 : 500, Abcam, ab9787), Romo-1 (1 : 200, AVIVA Systems Biology, ARP58431_P050), Iba-1 (1 : 1000, Abcam, ab5076), MPO (1 : 500, Abcam, ab65871); Lamin B1 (1 : 1000, Proteintech, 12987-1-AP), and *β*-actin (1 : 4000, Santa Cruz Biotechnology, sc-47778). Appropriate secondary antibodies (1 : 4000, Santa Cruz Biotechnology) were selected for the incubated membrane the following day for 1 h at room temperature. Immunoblots were then visualized with an ECL Plus chemiluminescence reagent kit (RPN3243; Amersham Bioscience, Bensenville, IL, USA) and quantified with optical methods using the ImageJ software (ImageJ 1.5; NIH, Bethesda, MD, USA). The results were normalized using *β*-actin or Lamin B1 as an internal control.

### 2.11. Statistical Analysis

All data were expressed as the mean and standard deviation (mean ± SD). Statistical analysis was performed with GraphPad Prism 6 software (La Jolla, CA, USA). Before analysis, the Shapiro-Wilk test was used to test normality. For parametric data, one-way ANOVA with post hoc Tukey test was used to test for differences among groups. *p* < 0.05 was considered statistically significant.

## 3. Results

### 3.1. Mortality and Exclusion

Of the 198 total animals used, 144 were subjected to MCAO, and the overall mortality was 16.7% (24/144). No significant difference was observed in mortality between the MCAO groups (*p* > 0.05). No rats died in the sham and naive groups. Six animals were excluded from this study due to no infarction volume after MCAO (Table 1).

### 3.2. Time-Course of Endogenous p-AMPK after MCAO

**Figure 2:**
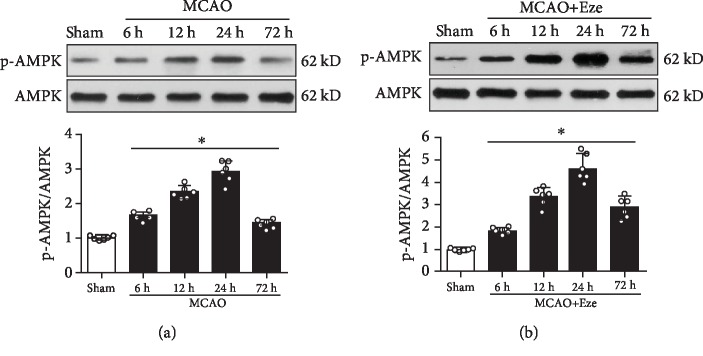
Expression of endogenous p-AMPK after MCAO. (a) Representative western blot bands and quantitative analysis of p-AMPK expression in the ipsilateral hemisphere after MCAO. (b) Representative western blot band and quantitative analysis of p-AMPK expression after treatment with Ezetimibe. The dose of Ezetimibe was 500 *μ*g/kg. ^∗^*p* < 0.05 vs. sham. *n* = 6 per group.

The expression of endogenous p-AMPK in the ipsilateral/right cerebral hemispheres after MCAO was assessed by western blot. As shown in Figure 2(a), the expression of p-AMPK increased at 6 h, reaching its peak at 24 h, and decreased by 72 h after MCAO compared to the sham group (*p* < 0.05). After Eze treatment, the endogenous p-AMPK expression further increased at 6, 12, 24, and 72 h after MCAO compared to the sham group (*p* < 0.05, Figure 2(b)).

### 3.3. Eze Treatment Reduced Brain Infraction and Ameliorated Neurobehavioral Deficiency at 24 h after MCAO

**Figure 3:**
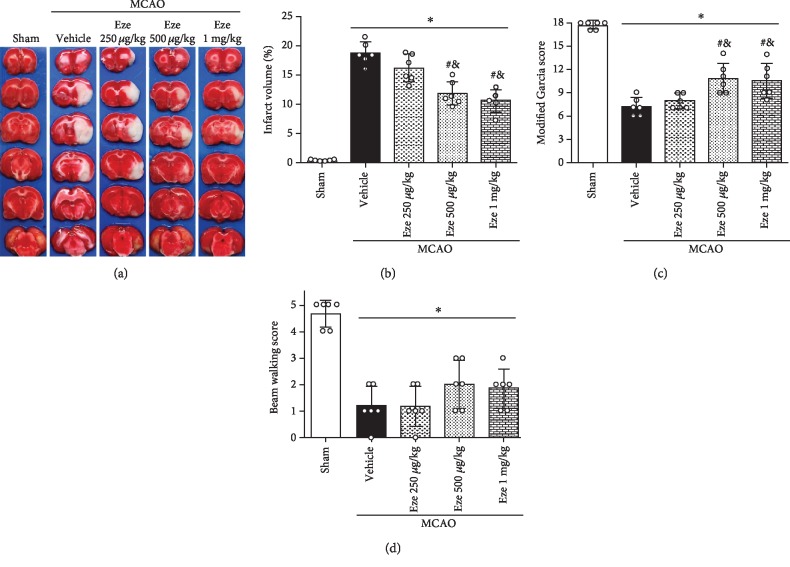
Eze reduced brain infraction and improved neurological outcomes at 24 h after MCAO. (a–c) Intranasal administration of Eze reduced infarction volume and improved the Garcia score at 24 h after MCAO. (d) There were no differences in beam walking scores between the MCAO groups. ^∗^*p* < 0.05 vs. sham, ^#^*p* < 0.05 vs. vehicle, and ^&^*p* < 0.05 vs. Eze 250 *μ*g/kg. *n* = 6 per group. Eze: Ezetimibe.

In the vehicle group, infarction volume was significantly increased, while Garcia scores were significantly decreased compared to the sham group (*p* < 0.05, Figure 3). Intranasally administered Eze 500 *μ*g/kg and Eze 1 mg/kg significantly reduced the infarction volume and improved neurological outcomes at 24 h after MCAO compared to the vehicle group (*p* < 0.05, Figures 3(a)–3(c)). With no additional therapeutic effects observed with Eze 1 mg/kg treatments, we used Eze 500 *μ*g/kg for the subsequent studies. There were no significant differences in beam walking scores between the MCAO groups (Figure 3(d)).

### 3.4. Eze Treatment Inhibited Neutrophil Infiltration and Microglia/Macrophage Activation at 24 h after MCAO

**Figure 4:**
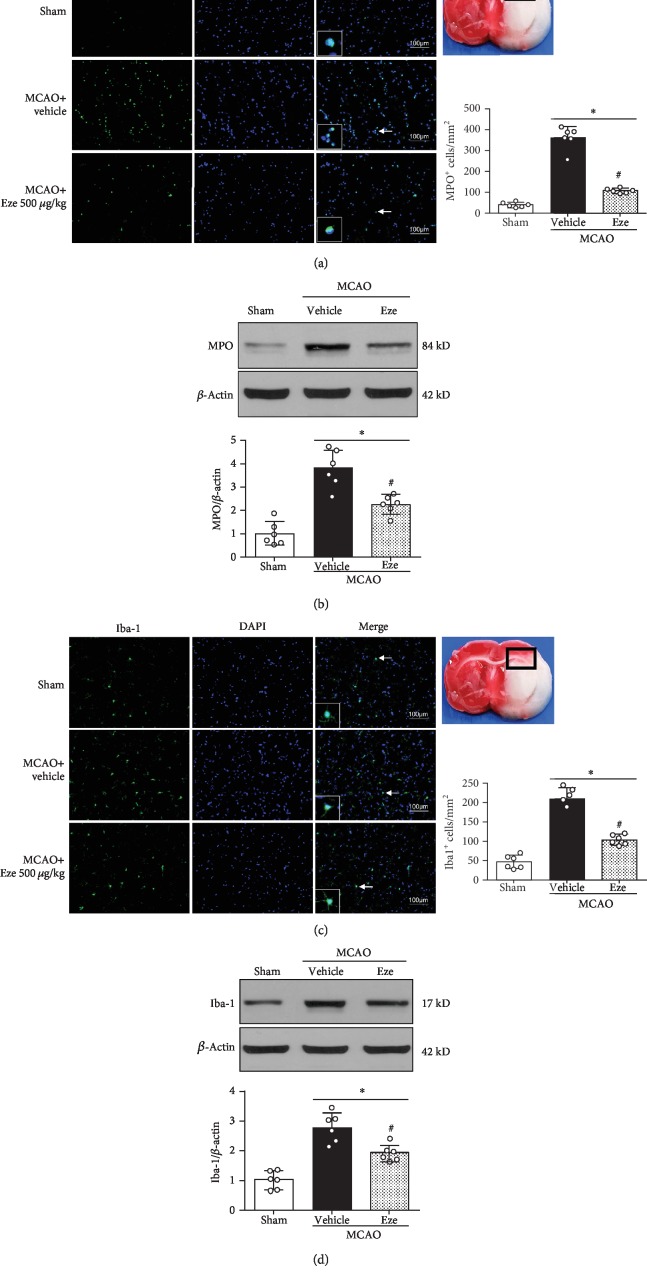
Eze inhibited microglia/macrophage activation and neutrophil infiltration at 24 h after MCAO. (a, c) Immunofluorescence revealed that treatment with Eze reduced the number of MPO-positive cells and Iba-1-positive cells in the ischemic penumbra region. (b, d) Representative western blot bands and quantitative analyses of MPO and Iba-1 protein levels at 24 h after MCAO. ^∗^*p* < 0.05 vs. sham; ^#^*p* < 0.05 vs. vehicle. *n* = 6 per group. Eze: Ezetimibe. Scale bar = 100 *μ*m.

MPO levels were used to assess neutrophil infiltration [8]. Iba-1 levels were used to evaluate microglia/macrophage activation in the brain tissue [42]. Immunofluorescence staining and western blot were used to evaluate whether the anti-inflammatory effects of Eze were caused by a reduction of neutrophil infiltration or microglia/macrophage activation in the ischemic penumbra at 24 h after MCAO. The immunofluorescence staining results showed that Eze treatment significantly decreased the number of MPO and Iba-1-positive cells in the ischemic penumbra compared to the vehicle group (*p* < 0.05, Figures 4(a) and 4(c)). Paralleling these findings, western blot results reported a decrease in expression of MPO and Iba-1 in the ipsilateral hemisphere after Eze treatment compared to the vehicle group (*p* < 0.05, Figures 4(b) and 4(d)).

### 3.5. Eze Treatment Reduced Oxidative Stress Injury at 24 h after MCAO

**Figure 5:**
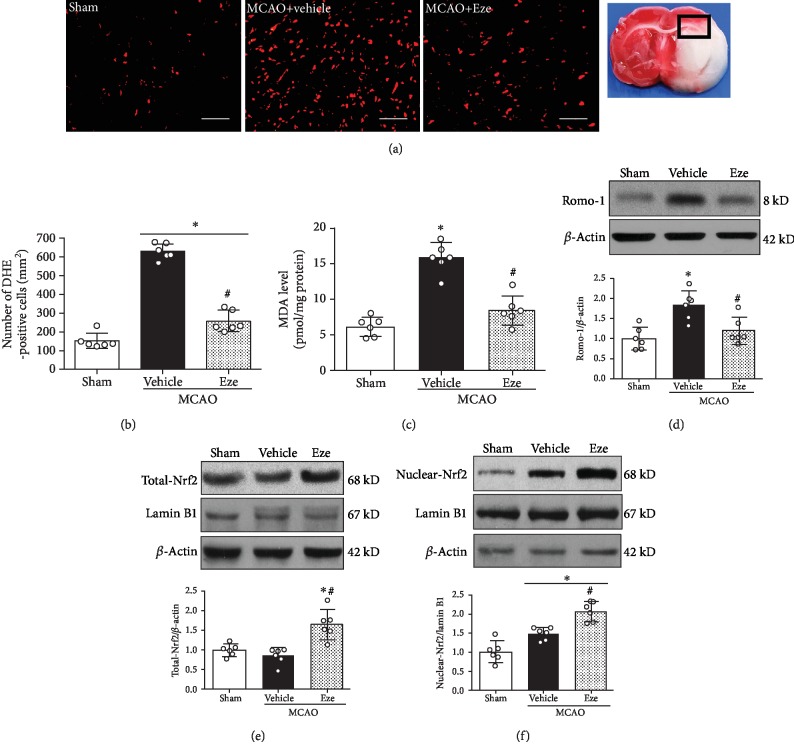
Eze reduced oxidative stress injury at 24 h after MCAO. (a) Representative microphotograph of DHE staining in the ischemic penumbra region. (b) Quantitative analysis of DHE-positive cells in the ischemic penumbra region. (c) The MDA level in the ischemic penumbra region. (d) Representative western blot band and quantitative analysis of Romo-1 expression. (e, f) Representative western blot bands and quantitative analyses of total-Nrf2 and nuclear-Nrf2 expression. ^∗^*p* < 0.05 vs. sham; ^#^*p* < 0.05 vs. vehicle. *n* = 6 per group. DHE: dihydroethidium; Eze: Ezetimibe; MDA: malonaldehyde. Scale bar = 100 *μ*m.

Oxidative stress levels of the ipsilateral hemisphere after MCAO were measured by MDA levels, DHE staining, and Romo-1 expression. At 24 h after MCAO, DHE-positive cells and MDA levels were higher in the vehicle group compared to the sham group (*p* < 0.05, Figures 5(a)–5(c)); however, after intranasal administration of Eze, DHE-positive cells and MDA levels were significantly reduced compared to the vehicle group (*p* < 0.05, Figures 5(a)–5(c)). In the vehicle group, Romo-1, a marker of oxidative stress [43], was increased compared to that in the sham group (*p* < 0.05, Figure 5(d)), while treatment with Eze significantly reduced the expression of Romo-1 at 24 h after MCAO (*p* < 0.05, Figure 5(d)). Nrf2 is a transcription factor involved in the endogenous antioxidant stress system [26]. Our results showed no significant difference in total-Nrf2; however, the nuclear-Nrf2 expression increased in the vehicle group compared to the sham group (Figures 5(e) and 5(f)). Treatment with Eze significantly increased total-Nrf2 expression and further increased the expression of nuclear-Nrf2 at 24 h after MCAO compared to the vehicle group (*p* < 0.05, Figures 5(e) and 5(f)).

### 3.6. Dorsomorphin and Nrf2 siRNA Abolished the Neuroprotective Effects of Eze at 24 h after MCAO

**Figure 6:**
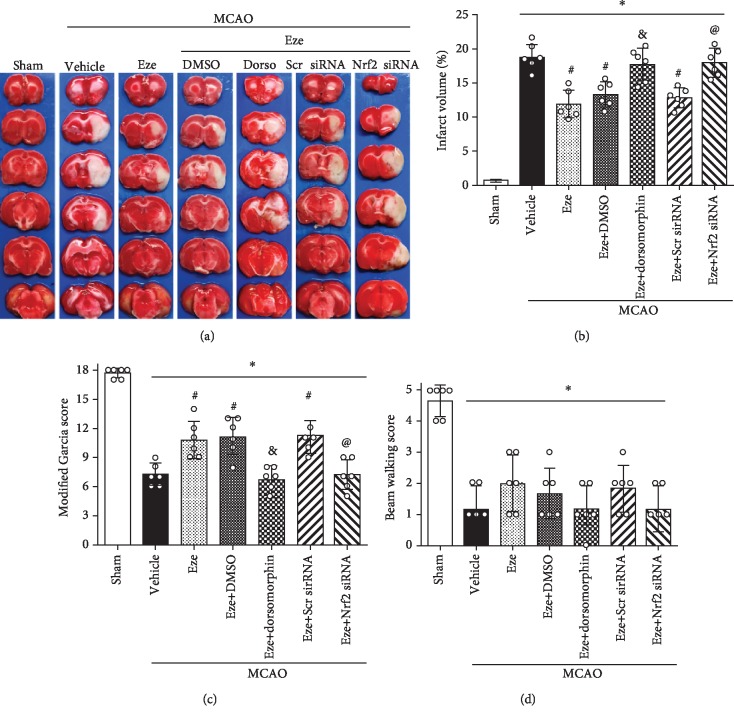
Dorsomorphin and Nrf2 siRNA reversed the neuroprotective effects of Eze after MCAO. (a) Representative image of TTC-staining brain slices, (b) quantified infarction volumes, (c) modified Garcia scores, and (d) beam walking scores at 24 h after MCAO. ^∗^*p* < 0.05 vs. sham, ^#^*p* < 0.05 vs. vehicle, ^&^*p* < 0.05 vs. Eze+DMSO, and ^@^*p* < 0.05 vs. Eze+Scr siRNA. *n* = 6 per group. Dorso: dorsomorphin; Eze: Ezetimibe; Scr siRNA: scramble siRNA.

Treatment with Eze reduced infarction volume and increased Garcia scores at 24 h after MCAO; however, this effect was reversed with the administration of both dorsomorphin (*p* < 0.05, Figures 6(a)–6(c)) and Nrf2 siRNA (*p* < 0.05, Figures 6(a)–6(c)). No significant differences were observed in beam walking scores between MCAO groups (Figure 6(d)).

### 3.7. Eze Treatment Attenuated Oxidative Stress and Neuroinflammation via Activation of the AMPK/Nrf2/TXNIP Pathway after MCAO in Rats

In the naive rat, the endogenous p-AMPK expression was significantly increased in the ipsilateral cortex after Eze treatment. Inhibition of AMPK with dorsomorphin significantly decreased the expression of p-AMPK in both naive and Eze-treated animals (*p* < 0.05, [Supplementary-material supplementary-material-1]).

**Figure 7:**
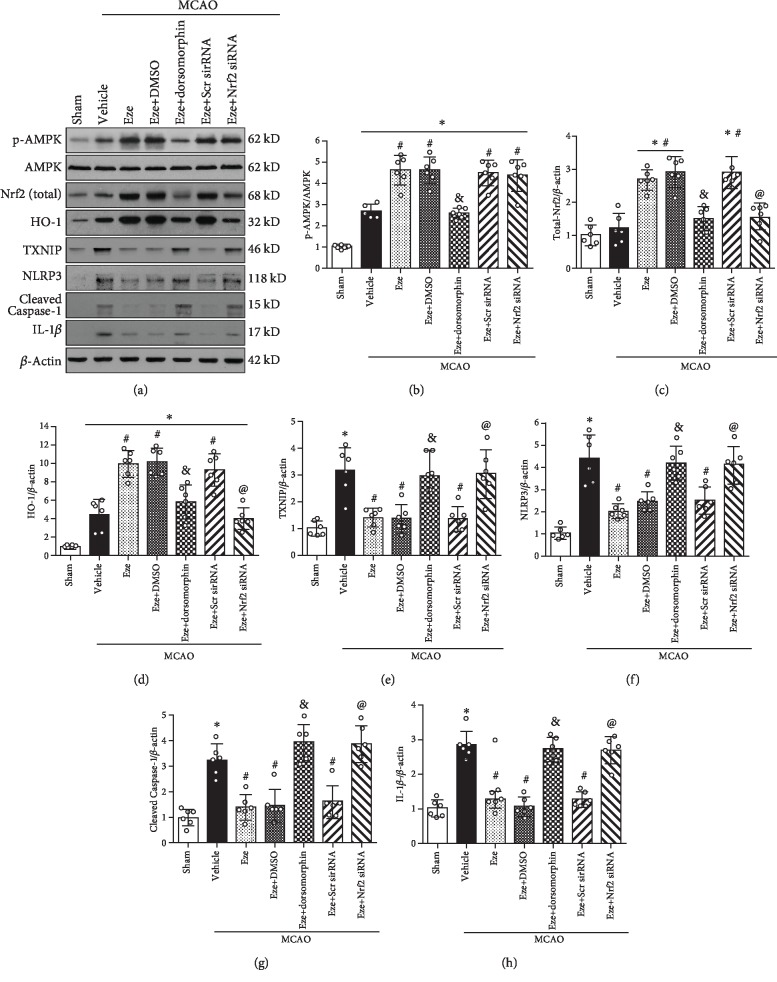
Eze attenuated oxidative stress and neuroinflammation via the AMPK/Nrf2/TXNIP pathway after MCAO. (a) Representative western blot bands. (b–h) Quantitative analyses of p-AMPK, Nrf2, HO-1, TXNIP, NLRP3, Cleaved Caspase-1, and IL-1*β* in the ipsilateral hemisphere at 24 h after MCAO. ^∗^*p* < 0.05 vs. sham, ^#^*p* < 0.05 vs. vehicle, ^&^*p* < 0.05 vs. Eze+DMSO, and ^@^*p* < 0.05 vs. Eze+Scr siRNA. *n* = 6 per group. Eze: Ezetimibe; Scr siRNA: scramble siRNA.

In the vehicle group at 24 h, the expression of p-AMPK, HO-1, TXNIP, NLRP3, Cleaved Caspase-1, and IL-1*β* increased compared to that in the sham group (*p* < 0.05, Figures 7(a) and 7(b) and 7(d)–7(h)). Intranasal administration of Eze increased the protein expression of total-Nrf2 and further increased p-AMPK and HO-1, while TXNIP, NLRP3, Cleaved Caspase-1, and IL-1*β* were reduced compared to that in the vehicle group (*p* < 0.05, Figures 7(a)–7(h)). However, inhibition of AMPK with dorsomorphin significantly decreased the protein expression of p-AMPK, Nrf2, and HO-1, while TXNIP, NLRP3, Cleaved Caspase-1, and IL-1*β* were increased compared to that in the MCAO+Eze+DMSO group at 24 h after MCAO (*p* < 0.05, Figures 7(a)–7(h)). Consistently, knockdown of endogenous Nrf2 with Nrf2 siRNA significantly decreased the protein expression of Nrf2 and HO-1, while TXNIP, NLRP3, Cleaved Caspase-1, and IL-1*β* were increased compared to that in the MCAO+Eze+Scr siRNA group at 24 h after MCAO (*p* < 0.05, Figures 7(a) and 7(c)–7(h)).

## 4. Discussion

**Figure 8:**
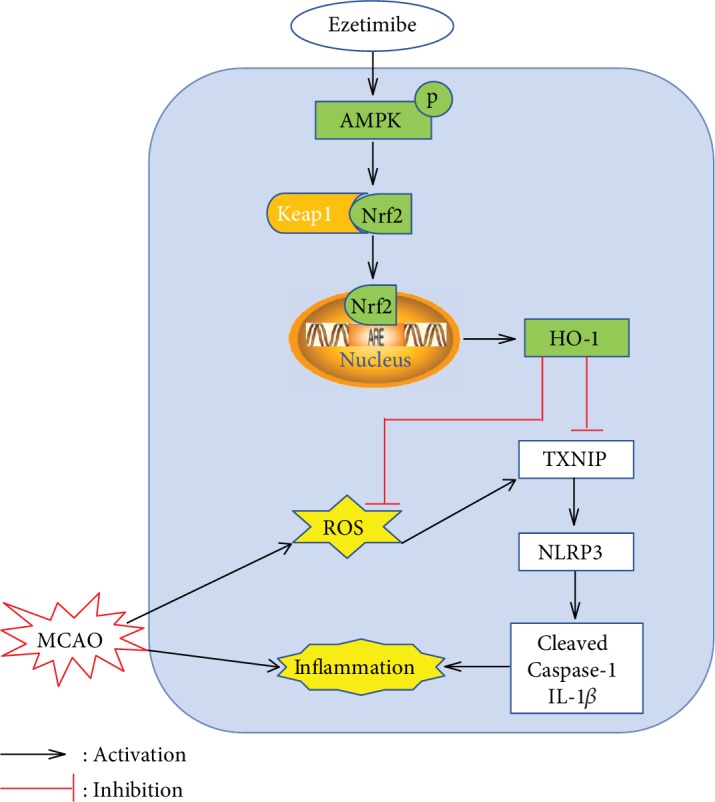
Proposed pathway for Eze and its downstream targets. Eze increases levels of phosphorylated AMPK and activates Nrf2, which translocates into the nucleus to increase the transcription of HO-1—a protein that inhibits ROS and inflammation.

In the present study, we demonstrated that Eze attenuated oxidative stress and neuroinflammation after MCAO. We made the following novel observations: (1) Eze further increased the endogenous p-AMPK expression after MCAO; (2) intranasal administration of Eze significantly reduced the infarction volume and improved neurological outcomes after MCAO; (3) Eze treatment inhibited neutrophil infiltration, microglia/macrophage activation, and oxidative stress-associated injuries in the ischemic penumbra regions after MCAO; (4) the antioxidative stress and anti-inflammatory effects of Eze were facilitated through the increased expression of p-AMPK, Nrf2, and HO-1, while Romo-1, TXNIP, NLRP3, Cleaved Caspase-1, and IL-1*β* were reduced following MCAO; and (5) pretreatment with dorsomorphin and Nrf2 siRNA reversed the beneficial effects of Eze on brain infarction, neurobehavioral function, and inflammatory protein expression. Taken together, our findings suggest that Eze attenuated oxidative stress and neuroinflammatory sequelae of MCAO via activation of the AMPK/Nrf2/TXNIP signaling pathway (Figure 8).

Accumulating scientific evidence suggests that neuroinflammation and oxidative stress are the main pathological processes responsible for the impairment of neurological function in MCAO [26, 29, 44, 45]. Clinically, Eze, a NPC1L1 inhibitor, is mainly used as a treatment for hypercholesterolemia; however, in addition to its lipid-lowering activity, several studies have reported that Eze may attenuate ischemic-related oxidative stress and inflammation [14, 19]. For example, in a rat liver ischemia/reperfusion model, Eze was reported to attenuate oxidative radicals, modulate NO production, and increase eNOS activity [13]. Independent of cholesterol regulation, other studies have shown that treatment with Eze improved renal injury outcomes in nondiabetic chronic kidney disease patients with dyslipidemia, which may be explained by the asymmetric dimethylarginine-lowering and antioxidative effects of Eze [46]. In a clinical study evaluating the neurological deterioration after embolic stroke resulting from atrial fibrillation in older patients, Lappegard et al. demonstrated that anti-inflammatory therapy with Eze may ameliorate the deterioration of neurocognitive function and loss of volume in cerebral areas [47]. Consistent with these findings, we demonstrated that treatment with Eze reduced brain infarction, neutrophil infiltration, microglia/macrophage activation, MDA levels, and DHE-positive cell numbers. Specifically, Eze treatment reduced the protein expression of Romo-1 (oxidative stress marker) and IL-1*β* (inflammatory marker). Congruently, neurological outcomes were improved after Eze administration at 24 h after MCAO.

Given that studies have reported Eze treatment to increase AMPK phosphorylation, novel and unique mechanistic endpoints are being investigated around AMPK regulation [16, 19, 20]. According to literature, Eze increases the oxygen consumption rate (OCR) and decreases the amount of ATP [48], which causes an elevation of ADP/ATP ratio that subsequently activates AMPK [20]. Therefore, Eze maintains cellular energy by regulating ATP consumption and generation via phosphorylation of AMPK [19]. Confirming this mechanistic link, our results showed that the endogenous p-AMPK expression was acutely increased after MCAO, and it was further increased after Eze treatment. To confirm that Eze regulates AMPK, a specific inhibitor of AMPK was administered with Eze. This intervention reversed the neuroprotective effects of Eze, increasing the infraction volume and neurobehavioral deficits; reducing the expression of p-AMPK, Nrf-2, and HO-1; and upregulating the expression of TXNIP, NLRP3, Cleaved Caspase-1, and IL-1*β*. This mechanistic study confirms a link between Eze and AMPK.

Nrf2 has traditionally been involved in upregulating antioxidant systems to reduce oxidative stress in the brain [49]. Under oxidative stress conditions, Nrf2 dissociates from Kelch-like ECH-associated protein 1 (Keap1) and translocates into the nucleus to bind to antioxidant response elements (ARE), which activates downstream antioxidant defense enzymes, such as HO-1 [50]. NLRP3 is an inflammasome protein complex located in the cell that binds to pro-Caspase-1 to cause neuronal apoptosis and inflammation in ischemic injuries [51]. Activation of AMPK and downstream inhibition of both NLRP3 inflammasomes and IL-1*β* mediates the anti-inflammatory effects of Eze [19]. Therefore, inhibition of NLRP3 inflammasomes via the AMPK pathway is neuroprotective in ischemic stroke [45]. Of importance, TXNIP, a redox-regulated protein, can bind to and activate the NLRP3 inflammasome in response to the oxidative stress associated with stroke [23], and when TXNIP is inhibited, Nrf2 acts as a negative regulator of the NLRP3 inflammasome [26]. Our results showed that Eze significantly enhanced the phosphorylation of AMPK; increased the expression of Nrf2 and HO-1; and subsequently decreased the expression of TXNIP, NLRP3, Cleaved Caspase-1, and IL-1*β*. Similarly, when knocking down the endogenous Nrf2 with Nrf2 siRNA, the protective effects of Eze were reversed: the intervention increased infarction volumes and neurobehavioral deficits, reducing the activation of Nrf2 and HO-1, with an associated increase in the levels of TXNIP, NLRP3, Cleaved Caspase-1, and IL-1*β*. Taken together, we can conclude that Eze exerts antioxidative and anti-inflammatory effects via activation of the AMPK/Nrf2/TXNIP signaling pathway.

There were some limitations in our study. First, due to the limited nature of this pilot study, we only evaluated a one-time window target for Eze treatment after MCAO. Second, our results do not fully exclude the possibility of alternative pathways that modulate the inflammasome pathway; thus, further research will need to investigate the relationship between other inflammasome activators (e.g., NF-*κ*B [52]) and the proposed pathway of Eze to fully exclude or incorporate alternative pathways. Finally, we have previously reported that Eze attenuates neuronal apoptosis via AMPK-induced autophagy [16]. In addition to these downstream targets, in the present study, Eze also decreased oxidative stress and subsequent neuroinflammation via activation of the AMPK/Nrf2/TXNIP pathway. Therefore, Eze may exert these effects by increasing the oxygen consumption rate, which then decreases the amount of ATP and phosphorylates AMPK, activating other downstream targets such as autophagy. However, since lowering cholesterol may also contribute to AMPK activation [16], NPC1L1 may have played a role in our proposed pathway. In sum, further research is needed to better understand the pleiotropic effects of Eze.

## 5. Conclusion

In summary, our findings suggest that intranasal administration of Eze reduced brain infarction, oxidative stress, and neuroinflammation, while neurological outcomes were improved after transient MCAO in rats. Mechanistically, the neuroprotective effects of Eze were mediated through activation of the AMPK/Nrf2/TXNIP pathway. This research supports the continued investigation of Eze as a potential therapy for the treatment of patients with ischemic stroke.

## Figures and Tables

**Table 1:** Summary of experimental groups and mortality rate in the study. A total of 198 rats were used in the following groups: naive (24), sham (24), and MCAO (150). Each experiment utilized a predefined exclusion criterion: no infarction volume after MCAO (*n* = 6).

Groups	Neuroscore TTC staining	IHC DHE	MDA	WB	Mortality	Exclusion	Subtotal
*Experiment 1*							
Sham				6	0	0	6
MCAO (6 h, 12 h, 24 h, and 72 h)				8∗6	10 (17.2%)	3	61
*Experiment 2*							
Sham	6	6	6	6	0	0	18
MCAO+vehicle	6	6	6	6	5 (21.7%)	1	24
MCAO+Eze (250 *μ*g/kg)	6				1 (14.3%)	0	7
MCAO+Eze (500 *μ*g/kg)	6	6	6	6	3 (14.3%)	0	21
MCAO+Eze (1 mg/kg)	6				1 (14.3%)	1	8
*Experiment 3*							
Naive+DMSO				6	0	0	6
Naive+dorsomorphin				6	0	0	6
Naive+Eze+DMSO				6	0	0	6
Naive+Eze+dorsomorphin				6	0	0	6
MCAO+Eze+DMSO	6			6	1 (14.3%)	0	7
MCAO+Eze+dorsomorphin	6			6	2 (25%)	0	8
MCAO+Eze+Scr siRNA	6			6	0	0	6
MCAO+Eze+Nrf2 siRNA	6			6	1 (14.3%)	1	8
Total	54	18	18	78 (+42)	24 (16.7%)	6	198

## Data Availability

All data are available upon request.

## Supplementary Materials

Supplementary Materials Figure S1: the effects of dorsomorphin on the expression of p-AMPK. Representative western blot bands and quantitative analysis of p-AMPK. ^∗^*p* < 0.05 vs. naive+DMSO, ^#^*p* < 0.05 vs. naive+Eze+DMSO, *n* = 6 per group.

## References

[1] GBD 2016 Causes of Death Collaborators (2017). Global, regional, and national age-sex specific mortality for 264 causes of death, 1980-2016: a systematic analysis for the Global Burden of Disease Study 2016. *The Lancet*.

[2] Moretti R., Pansiot J., Bettati D. (2015). Blood-brain barrier dysfunction in disorders of the developing brain. *Frontiers in Neuroscience*.

[3] Ozkul A., Akyol A., Yenisey C., Arpaci E., Kiylioglu N., Tataroglu C. (2007). Oxidative stress in acute ischemic stroke. *Journal of Clinical Neuroscience*.

[4] Chamorro A., Dirnagl U., Urra X., Planas A. M. (2016). Neuroprotection in acute stroke: targeting excitotoxicity, oxidative and nitrosative stress, and inflammation. *Lancet Neurology*.

[5] Shen M. H., Zhang C. B., Zhang J. H., Li P. F. (2016). Electroacupuncture attenuates cerebral ischemia and reperfusion injury in middle cerebral artery occlusion of rat via modulation of apoptosis, inflammation, oxidative stress, and excitotoxicity. *Evidence-based Complementary and Alternative Medicine*.

[6] Cai W., Liu S., Hu M. (2018). Post-stroke DHA treatment protects against acute ischemic brain injury by skewing macrophage polarity toward the M2 phenotype. *Translational Stroke Research*.

[7] Tian J., Guo S., Chen H. (2018). Combination of emricasan with ponatinib synergistically reduces ischemia/reperfusion injury in rat brain through simultaneous prevention of apoptosis and necroptosis. *Translational Stroke Research*.

[8] Yu G., Liang Y., Zheng S., Zhang H. (2018). Inhibition of myeloperoxidase by N-acetyl lysyltyrosylcysteine amide reduces oxidative stress-mediated inflammation, neuronal damage, and neural stem cell injury in a murine model of stroke. *The Journal of Pharmacology and Experimental Therapeutics*.

[9] Chen Y. J., Nguyen H. M., Maezawa I. (2016). The potassium channel KCa3.1 constitutes a pharmacological target for neuroinflammation associated with ischemia/reperfusion stroke. *Journal of Cerebral Blood Flow and Metabolism*.

[10] Roger V. L., Go A. S., Lloyd-Jones D. M. (2012). Heart disease and stroke statistics--2012 update: a report from the American Heart Association. *Circulation*.

[11] Altmann S. W., Davis H. R., Zhu L. J. (2004). Niemann-Pick C1 like 1 protein is critical for intestinal cholesterol absorption. *Science*.

[12] Garcia-Calvo M., Lisnock J., Bull H. G. (2005). The target of ezetimibe is Niemann-Pick C1-like 1 (NPC1L1). *Proceedings of the National Academy of Sciences of the United States of America*.

[13] Trocha M., Merwid-Ląd A., Sozański T. (2013). Influence of ezetimibe on ADMA-DDAH-NO pathway in rat liver subjected to partial ischemia followed by global reperfusion. *Pharmacological Reports*.

[14] Trocha M., Merwid-Lad A., Chlebda E. (2014). Influence of ezetimibe on selected parameters of oxidative stress in rat liver subjected to ischemia/reperfusion. *Archives of Medical Science*.

[15] Qin L., Yang Y. B., Yang Y. X. (2014). Inhibition of smooth muscle cell proliferation by ezetimibe via the cyclin D1-MAPK pathway. *Journal of Pharmacological Sciences*.

[16] Yu J., Li X., Matei N. (2018). Ezetimibe, a NPC1L1 inhibitor, attenuates neuronal apoptosis through AMPK dependent autophagy activation after MCAO in rats. *Experimental Neurology*.

[17] Dalla Y., Singh N., Jaggi A. S., Singh D., Ghulati P. (2009). Potential of ezetimibe in memory deficits associated with dementia of Alzheimer's type in mice. *Indian Journal of Pharmacology*.

[18] Yang L., Zhao P., Zhao J., Wang J., Shi L., Wang X. (2016). Effects of ezetimibe and anticoagulant combined therapy on progressing stroke: a randomized, placebo-controlled study. *Journal of Neurology*.

[19] Kim S. H., Kim G., Han D. H. (2017). Ezetimibe ameliorates steatohepatitis via AMP activated protein kinase-TFEB-mediated activation of autophagy and NLRP3 inflammasome inhibition. *Autophagy*.

[20] Lee D. H., Han D. H., Nam K. T. (2016). Ezetimibe, an NPC1L1 inhibitor, is a potent Nrf2 activator that protects mice from diet-induced nonalcoholic steatohepatitis. *Free Radical Biology & Medicine*.

[21] Park S. Y., Jin M. L., Ko M. J., Park G., Choi Y. W. (2016). Anti-neuroinflammatory effect of emodin in LPS-stimulated microglia: involvement of AMPK/Nrf2 activation. *Neurochemical Research*.

[22] Wu W. Y., Li Y. D., Cui Y. K. (2018). The natural flavone acacetin confers cardiomyocyte protection against hypoxia/reoxygenation injury via AMPK-mediated activation of Nrf2 signaling pathway. *Frontiers in Pharmacology*.

[23] Zhou R., Tardivel A., Thorens B., Choi I., Tschopp J. (2010). Thioredoxin-interacting protein links oxidative stress to inflammasome activation. *Nature Immunology*.

[24] Ishrat T., Mohamed I. N., Pillai B. (2015). Thioredoxin-interacting protein: a novel target for neuroprotection in experimental thromboembolic stroke in mice. *Molecular Neurobiology*.

[25] Li Y., Li J., Li S. (2015). Curcumin attenuates glutamate neurotoxicity in the hippocampus by suppression of ER stress-associated TXNIP/NLRP3 inflammasome activation in a manner dependent on AMPK. *Toxicology and Applied Pharmacology*.

[26] Hou Y., Wang Y., He Q. (2018). Nrf2 inhibits NLRP3 inflammasome activation through regulating Trx1/TXNIP complex in cerebral ischemia reperfusion injury. *Behavioural Brain Research*.

[27] Kilkenny C., Browne W. J., Cuthill I. C., Emerson M., Altman D. G. (2010). Improving bioscience research reporting: the ARRIVE guidelines for reporting animal research. *PLoS Biology*.

[28] Matei N., Camara J., McBride D. (2018). Intranasal wnt3a attenuates neuronal apoptosis through Frz1/PIWIL1a/FOXM1 pathway in MCAO rats. *The Journal of Neuroscience*.

[29] Wu G., McBride D. W., Zhang J. H. (2018). Axl activation attenuates neuroinflammation by inhibiting the TLR/TRAF/NF-*κ*B pathway after MCAO in rats. *Neurobiology of Disease*.

[30] An J. Y., Zhou L. L., Sun P. (2015). Role of the AMPK signaling pathway in early brain injury after subarachnoid hemorrhage in rats. *Acta Neurochirurgica*.

[31] Zhou K., Enkhjargal B., Xie Z. (2018). Dihydrolipoic acid inhibits lysosomal rupture and NLRP3 through lysosome-associated membrane protein-1/calcium/calmodulin-dependent protein kinase II/TAK1 pathways after subarachnoid hemorrhage in rat. *Stroke*.

[32] Garcia J. H., Wagner S., Liu K. F., Hu X. J. (1995). Neurological deficit and extent of neuronal necrosis attributable to middle cerebral artery occlusion in rats. *Stroke*.

[33] Wahl F., Allix M., Plotkine M., Boulu R. G. (1992). Neurological and behavioral outcomes of focal cerebral ischemia in rats. *Stroke*.

[34] Germano A. F., Dixon C. E., d'Avella D., Hayes R. L., Tomasello F. (1994). Behavioral deficits following experimental subarachnoid hemorrhage in the rat. *Journal of Neurotrauma*.

[35] Goldstein L. B., Davis J. N. (1990). Beam-walking in rats: studies towards developing an animal model of functional recovery after brain injury. *Journal of Neuroscience Methods*.

[36] Griemert E. V., Recarte Pelz K., Engelhard K., Schäfer M. K., Thal S. C. (2019). PAI-1 but not PAI-2 gene deficiency attenuates ischemic brain injury after experimental stroke. *Translational Stroke Research*.

[37] McBride D. W., Tang J., Zhang J. H. (2016). Development of an infarct volume algorithm to correct for brain swelling after ischemic stroke in rats. *Acta Neurochirurgica. Supplement*.

[38] Xu N., Zhang Y., Doycheva D. M. (2018). Adiponectin attenuates neuronal apoptosis induced by hypoxia-ischemia via the activation of AdipoR1/APPL1/LKB1/AMPK pathway in neonatal rats. *Neuropharmacology*.

[39] McBride D. W., Wu G., Nowrangi D. (2018). Delayed recanalization promotes functional recovery in rats following permanent middle cerebral artery occlusion. *Translational Stroke Research*.

[40] Wang H. W., Huang B. S., White R. A., Chen A., Ahmad M., Leenen F. H. (2016). Mineralocorticoid and angiotensin II type 1 receptors in the subfornical organ mediate angiotensin II - induced hypothalamic reactive oxygen species and hypertension. *Neuroscience*.

[41] Xie Z., Enkhjargal B., Wu L. (2018). Exendin-4 attenuates neuronal death via GLP-1R/PI3K/Akt pathway in early brain injury after subarachnoid hemorrhage in rats. *Neuropharmacology*.

[42] Chen S., Zhao L., Sherchan P. (2018). Activation of melanocortin receptor 4 with RO27-3225 attenuates neuroinflammation through AMPK/JNK/p38 MAPK pathway after intracerebral hemorrhage in mice. *Journal of Neuroinflammation*.

[43] Chung Y. M., Kim J. S., Yoo Y. D. (2006). A novel protein, Romo1, induces ROS production in the mitochondria. *Biochemical and Biophysical Research Communications*.

[44] Zhang S., Jiang L., Che F., Lu Y., Xie Z., Wang H. (2017). Arctigenin attenuates ischemic stroke via SIRT1-dependent inhibition of NLRP3 inflammasome. *Biochemical and Biophysical Research Communications*.

[45] Qiu J., Wang M., Zhang J. (2016). The neuroprotection of sinomenine against ischemic stroke in mice by suppressing NLRP3 inflammasome via AMPK signaling. *International Immunopharmacology*.

[46] Nakamura T., Sato E., Fujiwara N. (2009). Ezetimibe decreases serum levels of asymmetric dimethylarginine (ADMA) and ameliorates renal injury in non-diabetic chronic kidney disease patients in a cholesterol-independent manner. *Pharmacological Research*.

[47] Lappegard K. T., Pop-Purceleanu M., van Heerde W., Sexton J., Tendolkar I., Pop G. (2013). Improved neurocognitive functions correlate with reduced inflammatory burden in atrial fibrillation patients treated with intensive cholesterol lowering therapy. *Journal of Neuroinflammation*.

[48] Hernandez-Mijares A., Banuls C., Rovira-Llopis S. (2016). Effects of simvastatin, ezetimibe and simvastatin/ezetimibe on mitochondrial function and leukocyte/endothelial cell interactions in patients with hypercholesterolemia. *Atherosclerosis*.

[49] Narayanan S. V., Dave K. R., Perez-Pinzon M. A. (2018). Ischemic preconditioning protects astrocytes against oxygen glucose deprivation via the nuclear erythroid 2-related factor 2 pathway. *Translational Stroke Research*.

[50] McMahon M., Itoh K., Yamamoto M., Hayes J. D. (2003). Keap1-dependent proteasomal degradation of transcription factor Nrf2 contributes to the negative regulation of antioxidant response element-driven gene expression. *The Journal of Biological Chemistry*.

[51] Shaw P. J., McDermott M. F., Kanneganti T. D. (2011). Inflammasomes and autoimmunity. *Trends in Molecular Medicine*.

[52] Bauernfeind F., Ablasser A., Bartok E. (2011). Inflammasomes: current understanding and open questions. *Cellular and Molecular Life Sciences*.

