# Supplementary figures and images for: Ezetimibe Attenuates Oxidative Stress and Neuroinflammation via the AMPK/Nrf2/TXNIP Pathway after MCAO in Rats
Source: Oxid Med Cell Longev. 2020 Jan 3;2020:4717258. doi: 10.1155/2020/4717258 (PMC6964721; doi:10.1155/2020/4717258)

## Slide 1
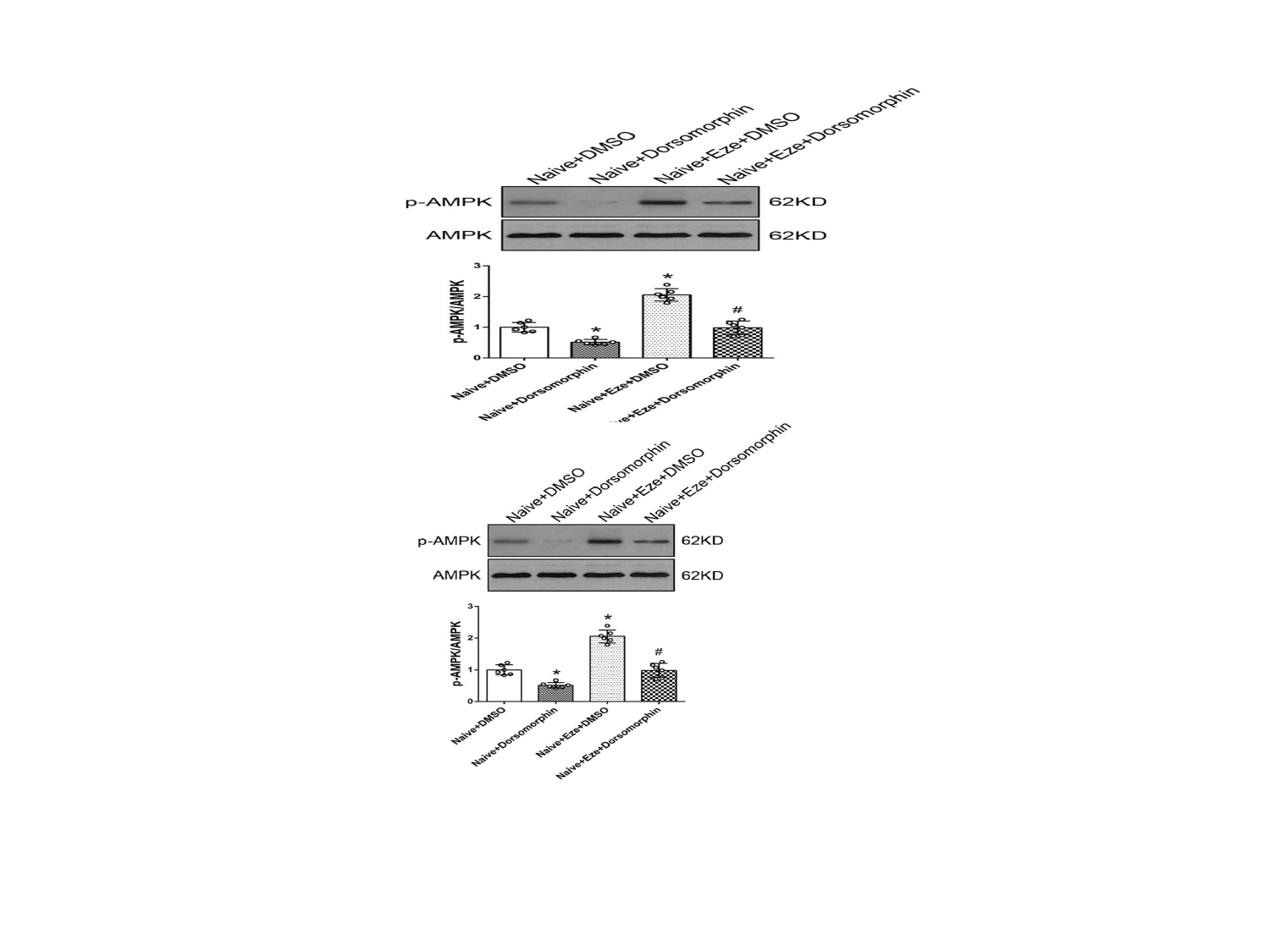

Supplement: Supplementary Materials — Figure S1: the effects of dorsomorphin on the expression of p-AMPK. Representative western blot bands and quantitative analysis of p-AMPK. ∗p < 0.05 vs. naive+DMSO, #p < 0.05 vs. naive+Eze+DMSO, n = 6 per group. [file 4717258.f1.ppt]
